# Cellular availability of *all-trans*-retinoic acid in mice: a pilot study

**DOI:** 10.1016/j.bbrep.2026.102839

**Published:** 2026-10-15

**Authors:** Masaharu Yoshihara, Yoshitoku Watabe, Baoshuo Ning, Hayate Suzuki, Seiya Mizuno, Satoru Takahashi

**Affiliations:** a Department of Anatomy and Embryology, Institute of Medicine, University of Tsukuba, 1-1-1, Tennodai, Tsukuba, Ibaraki, 305-8575, Japan; b College of Medicine, School of Medicine and Health Sciences, University of Tsukuba, 1-1-1, Tennodai, Tsukuba, Ibaraki, 305-8575, Japan; c Laboratory Animal Resource Center, Transborder Medical Research Center, Institute of Medicine, University of Tsukuba, 1-1-1, Tennodai, Tsukuba, Ibaraki, 305-8575, Japan

**Keywords:** Clinical nutrition, Cre/loxP system, Developmental biology, Gal4/UAS system, Gene regulation, Retinoic acid signaling

## Abstract

Retinoic acid signaling is essential for mammalian development and health. The genetic manipulations that enable visualization of this signal have revealed its involvement in many organs, including the eyes, colon, and trunk regions of mouse embryos. The *in vivo* cellular availability of the ligand, *all-trans-*retinoic acid (ATRA), is, however, still unclear, even though it, along with receptor expression, constitutes an essential part of signal transduction. In this pilot study, we sought to identify cells with ATRA availability by introducing a fusion protein comprising the mouse retinoic acid receptor alpha ligand-binding domain and the yeast Gal4 DNA-binding domain in mice. By crossing with other reporter mice with Gal4-responsive Cre expression and subsequent tandem DsRed expression to track ATRA availability through adulthood, we labeled the *in vivo* cellular availability of ATRA in two out of 14 mice from three F1-generation lines. As expected, we observed prior ATRA availability in organs associated with this signaling pathway, including the eye and colon. In addition, we observed prior ATRA availability in gastric epithelial cells and hepatocytes, which originates from the intestinal region where ATRA availability has not been determined, suggesting that ATRA's involvement in development or physiology may be greater than previously thought. Unfortunately, prior ATRA availability was limited to a small population of cells in the animals owing to low expression of the fusion receptor, as determined by quantitative reverse-transcription PCR. In summary, this pilot study suggested extensive effects of ATRA, underscoring its potential importance in developmental biology and clinical nutrition. In addition, this study encourages careful transgene design by reporting our experience with a low success rate in transgene expression.

## Highlights

• Gal4-fused retinoic receptor functioned *in vivo.*
• Prior ATRA availability was labeled *in vivo.*
• ATRA availability in unreported cells was suggested.

## 1 Introduction

Retinoic acid signaling (RA signaling) plays multiple roles from the embryonic stage to adulthood in mammals. For example, it is associated with the development of many organs, including the eye [1] and colon [2] [3], and with the physiological maintenance of the eye and gastrointestinal tract, since its deficiency causes eye symptoms and diarrhea [4]. In the colon, RA levels were also associated with the progression of human and mouse colitis and colorectal cancers [4]. The physiologically active ligand molecule (*all-trans-*retinoic acid, ATRA) is derived from retinol (Vitamin A) in two enzymatic steps and is inactivated by CYP enzymes [1]. In the absence of ATRA, retinoic acid receptors (RARα, RARβ, RARγ) are bound with multiple co-repressors. ATRA releases these co-repressors to induce gene expression under the retinoic acid-responsive element (RARE). Notably, retinoic acid receptors have their own DNA-binding domain (DBD) apart from their ligand-binding domain (LBD) [5]. This resembles a yeast transcription factor, Gal4, which has its DBD and activation domain at the N- and C-termini, respectively [6].

RA signaling has been detected using a transgenic mouse in which β-galactosidase is expressed under the control of a RARE (RARE-lacZ mice) [7]. How signaling is regulated *in vivo*, however, remains unclear, as the timing and location of ligand availability have not been documented despite many attempts to estimate them by visualizing ATRA-producing or ATRA-inactivating enzymes [1]. The embryonic lethal phenotypes in knockout mice of ATRA-synthesizing or -catalyzing genes also make it difficult to elucidate the regulation of RA signaling from the embryonic stage to adulthood [5]. To directly observe ATRA availability at single-cell resolution, we attempted to generate transgenic mice expressing a fusion protein comprising the RARα ligand-binding domain (RaraLBD) and the Gal4 DNA-binding domain (Gal4DBD), which has been shown to induce Gal4-dependent gene expression in response to ATRA *in vitro* [8]. We crossed these mice with two transgenic lines that comprise a Gal4-responsive Cre expression element (UAS-Cre mice) [9] and a Cre-dependent tandem DsRed (tDsRed) reporter element (R26GRR mice) [10] (Fig. 1). In this system, prior and ongoing ATRA availabilities are labeled with tDsRed and Cre, respectively. Here, we report tDsRed observations in two of 14 mice across three F1-generation lines. We observed tDsRed expression in both well-documented cells (e.g., retinal cells and cells in the muscle layer of the colon) and obscure cells (e.g., gastric epithelial cells and hepatocytes), suggesting a broader involvement of this signaling in development or physiology than previously considered.

**Fig. 1:**
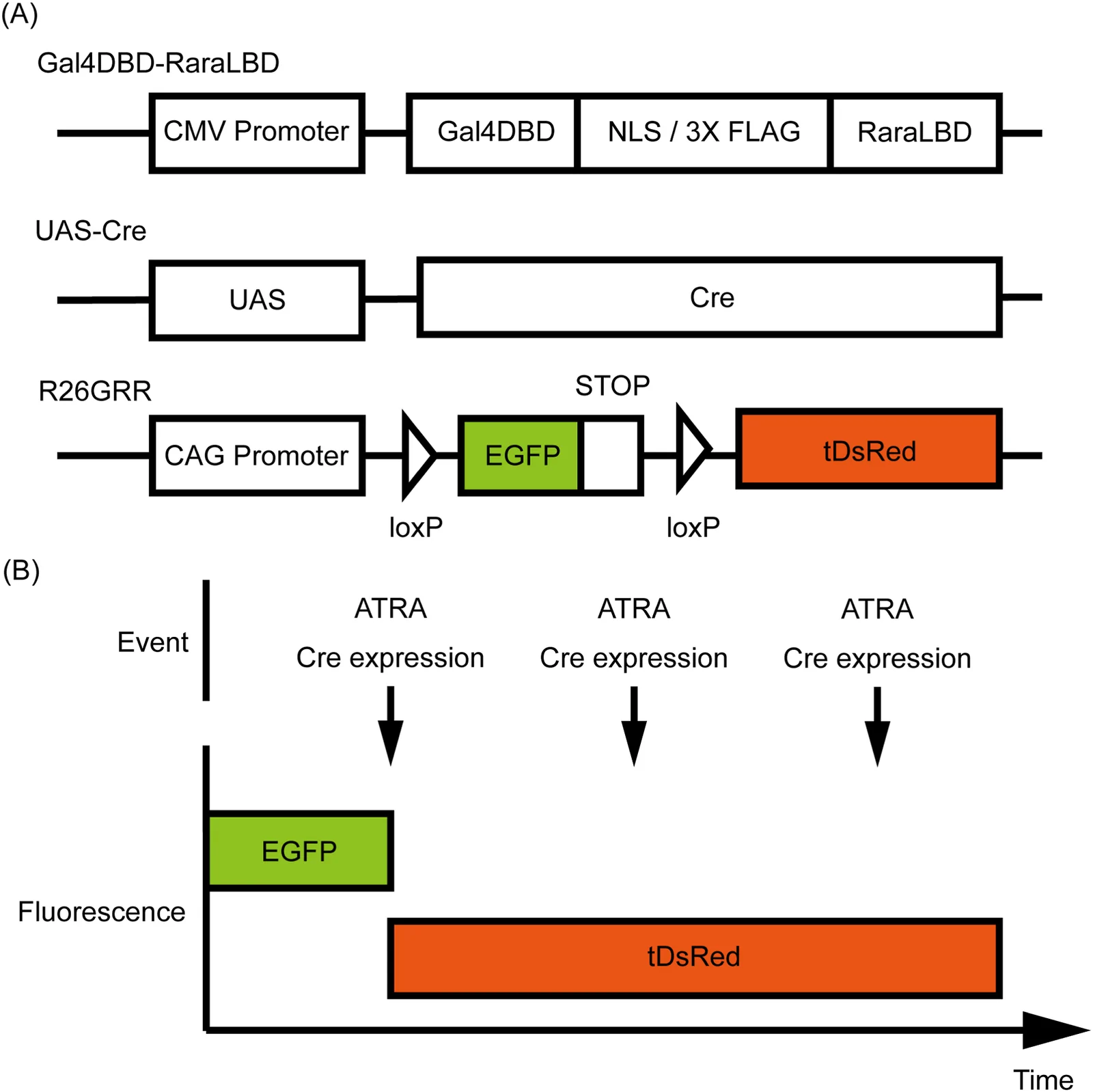
System for labeling cellular ATRA availability. (A) The three transgenic mouse lines used in this study. The Gal4DBD-RaraLBD mice were newly generated in this study. Note that we showed only essential gene elements to understand the experimental strategy. In addition, the CMV IE enhancer/promoter is referred to as the “CMV promoter” for brevity. NLS: nuclear localization sequence. (B) Before ATRA binds to Gal4DBD-RaraLBD, EGFP is expressed. The binding of ATRA and Gal4DBD-RaraLBD induces expression of Cre recombinase (ongoing ATRA availability), which converts fluorescence from EGFP to tDsRed (prior ATRA availability). Note that binding of ATRA and Gal4DBD-RaraLBD could occur multiple times.

## 2 Materials and methods

### 2.1 pcDNA3_Gal4DBD-RaraLBD construction

For Gal4DBD, we used the first 147 amino acids [6] of the *Saccharomyces cerevisiae* S288C strain acquired from the Saccharomyces genome database (https://www.yeastgenome.org/locus/S000006169/sequence). We planned to use (1) CMV immediate early (IE) enhancer and promoter, human β-globin/IgG chimeric intron and Kozak sequence in its N-terminus, and (2) SV40 nuclear localization sequence (NLS), 3× FLAG sequence between Gal4DBD and RaraLBD, and (3) bovine growth hormone poly (A) sequence (bGH pA) in its C-terminus (Supplementary Material 1). The fusion sequence of human β-globin/IgG chimeric intron, Gal4DBD, SV40 NLS, 3× FLAG, and RaraLBD was supplied in IDT Vector Amp Blunt by Integrated DNA Technologies (Tokyo, Japan). The sequence from the human β-globin/IgG chimeric intron to RaraLBD was excised with EcoR1 and Not1 and then inserted into the pcDNA3 vector containing the CMV IE enhancer and promoter and the bGH pA. Hereafter, we refer to this plasmid as pcDNA3_Gal4DBD-RaraLBD. We confirmed the correct transgene sequence by direct sequencing (Fasmac, Kanagawa, Japan) with a CMV forward primer (5′-CGCAAATGGGCGGTAGGCGTG-3′) and an SP6 primer (5′-ATTTAGGTGACACTATAG-3′). The DNA sequence in the WORD file format is available in Supplementary Material 2. An ApE file for the DNA sequence is available upon request to the corresponding author.

### 2.2 In vitro experiments

HEK293T cells were cultured using 35 mm glass bottom dishes (Cat #: D11134H, Matsunami Glass, Osaka, Japan) in DMEM (high glucose) (Cat #: 11,960-044, Gibco, Grand Island, NY, USA) supplemented with fetal bovine serum (final concentration: 10%v/v) (Cat #: 556-33865, Fujifilm Wako, Osaka, Japan) and Penicillin-Streptomycin (final concentration: 100 U/mL) (Cat #: 15,140-148, Gibco). We transfected the cells with pcDNA3_Gal4DBD-RaraLBD and UAS-Cre plasmid (Addgene Plasmid #: 193,581) [9] using FuGENE HD Transfection Reagent (Cat #: E2311, Promega, Madison, WI, USA). The next day, we administered either 30 nM *all-trans*-retinoic acid (Cat #: 186-01114, Fujifilm Wako) [11] or vehicle (DMSO) (Cat #: 13,408-64, Nacalai Tesque, Kyoto, Japan) to the medium and cultured for another day. The cells were fixed with 4% Paraformaldehyde Phosphate Buffer Solution (Cat #: 09154-85, Nacalai Tesque), stained for Cre recombinase using rabbit monoclonal *anti*-Cre recombinase antibody (clone: D7L7 L, Cat #: 15036 S, RRID: AB_2798694, Cell Signaling Technology, Danvers, MA, USA) (1:500 dilution, 1 h, room temperature) and goat anti-rabbit IgG (H + L) highly cross-adsorbed secondary antibody, Alexa Fluor Plus 555 (Cat #: A32727, RRID: AB_2633276, ThermoFisher Scientific, Waltham, MA, USA) (1:1000 dilution, 1 h, room temperature), counterstained with Hoechst 33,342 (Cat: 346-07951, Fujifilm Wako) (final concentration: 1 μg/mL, 10 min, room temperature) and photographed using BIOREVO-BZ-X810 (Keyence, Osaka, Japan). We repeated this experiment three times and compared cell numbers in high-power fields using a chi-square test.

### 2.3 Animal experiments

We digested pcDNA3_Gal4DBD-RaraLBD with Pvu1 to obtain the linearized transgene. The transgenic mice (Gal4DBD-RaraLBD) were generated in the ICR background, essentially as in a previous report [9]. F0 generation mice were mated from wild-type ICR mice (Charles River Laboratories Japan, Kanagawa, Japan), and F1 generation mice were examined for germline transmission of the transgene by PCR using BIOTAQ DNA polymerase (Cat #: BIO-21040, Meridian Biosciences, Cincinnati, OH, USA) (Forward primer: 5′-AGTTGACTGTATCGCCAAAGA-3′, Reverse primer: 5′-CTCCACAGTCTTAATGATGCACT-3′, Annealing temperature: 55°, Product length: 217 base pairs). Among the multiple transgenic mouse lines, we chose a single F1-generation line (line 1711) for the initial analysis because it was the first to successfully mate with other reporter mice, and also examined two additional lines (lines 1679 and 1727). Analysis was conducted in mice after the F2 generation. For immunohistochemistry, we prepared formalin-fixed and paraffin-embedded sections from Gal4DBD-RaraLBD; UAS-Cre; R26GRR mice, carried out heat-induced epitope retrieval using DAKO Target Retrieval Solution (Cat #: S1699, Agilent Technologies, Santa Clara, CA, USA) (105°, 5 min), and incubated the samples with a rabbit polyclonal *anti*-DsRed antibody (Cat #: 600-401-379, Lot #: 46,317, RRID: AB_2209751, Rockland Immunochemicals, Philadelphia, PA, USA) (1:500 or 1:1000 dilution, 1.5 h, room temperature), Histofine Simple Stain Mouse MAX-PO (R) (Cat #: 414,341, Nichirei Biosciences, Tokyo, Japan) (room temperature, 30 min), DAB Substrate Kit (Cat #: 425,011, Nichirei Biosciences), and Mayer's Hematoxylin Solution (Cat # 131-09665, Fujifilm Wako). Images were acquired with BIOREVO-BZ-X810. We adjusted brightness, but not contrast, in Adobe Photoshop.

For the quantitative reverse transcription (RT)-PCR experiment, RNA from the eye and colon of adult Gal4DBD-RaraLBD or wild-type male mice (n = 3 for each group, 9-47 weeks old) was purified using Isogen (Cat #: 317-02503, Fujifilm Wako), and cDNA was prepared using ReverTra Ace qPCR RT Master Mix with gDNA Remover (Cat #: FSQ-301, Toyobo, Osaka, Japan). We used Thunderbird Next SYBR qPCR Mix (Cat #: QPX-201, Toyobo) and the Takara Thermal Cycler Dice Real-Time System (Takara Bio, Shiga, Japan) for RT-PCR. We used two primer sets: one for Gal4DBD-RaraLBD (Forward primer: 5′-AGAAAGGTTGGAGGCGGATC-3′, Reverse primer: 5′-CCTTGTCATCGTCATCCTTGT-3′) and one for the housekeeping gene HPRT (Forward primer: 5′-TTGTTGTTGGATATGCCCTTGACTA-3′, Reverse primer: 5′-AGGCAGATGGCCACAGGACTA-3′) [12].

### 2.4 Approval of DNA and animal experiments

Animal experiments were conducted in accordance with institutional and national guidelines. Approval was obtained from the Institutional Animal Care and Use Committee and the DNA Experiment Committee of the University of Tsukuba (Approval Numbers for Animal Experiments: 24-411, 25-160, 26-307) (Approval Number for DNA Experiments: 240208).

## 3 Results

### 3.1 Transgene functionality

We briefly checked transgene functionality *in vitro* before injection into mouse zygotes. HEK293T cells were transfected with the Gal4DBD-RaraLBD and UAS-Cre plasmids and treated with either 30 nM ATRA or vehicle (DMSO). Although the UAS-Cre plasmid contained a fluorescent protein, miRFP670, its fluorescence was too dim to detect, warranting signal enhancement by immunofluorescence. Cre expression was observed in 175 cells out of 13,113 Hoechst 33,342-positive cells (1.33%) in the ATRA group, while in 79 cells out of 16,600 Hoechst 33,342-positive cells in the DMSO group (0.48%) (p < 0.001 b y chi-square test), suggesting that the transgene is functional (Representative images are available in Supplementary Material 3). Although we did not perform a detailed binding-affinity analysis, we confirmed a statistically significant increase in Cre expression at a previously reported physiological ATRA level [11]. As the sensitivity for ATRA detection should depend on several factors, including the expression levels of Gal4DBD-RaraLBD proteins, we considered that final validation should be conducted *in vivo*.

### 3.2 ATRA availability *in vivo*

To maximize the chance of labeling ATRA availability, we permanently labeled prior ATRA availability using Cre recombinase (Fig. 1). We examined tDsRed expression in adult mice, focusing on the eye and colon, where ATRA is suggested to act during embryogenesis and adulthood. We observed tDsRed expression in some retinal (Fig. 2A and A’, 2 B, and 2 B′) and colonic (Fig. 2C and C’, 2D, and 2D’) cells in two mice (Animal IDs: 2092 and 2111) from the same F1 founder (line 1711). In the colon, we observed tDsRed expression in the muscle layer cells as well as in epithelial cells, whereas previous studies have suggested ATRA action in neurons [2] and immune cells [3]. Given RA is a diffusible molecule, our results were consistent with previous studies, suggesting that tDsRed signal reflects prior ATRA availability, albeit with low efficacy. Unfortunately, we did not observe Cre recombinase expression (ongoing ATRA availability) in these samples. This incompleteness is due to low expression of the Gal4DBD-RaraLBD protein, as Gal4DBD-RaraLBD mRNA was not detected by RT-PCR (Supplementary Material 4).

**Fig. 2:**
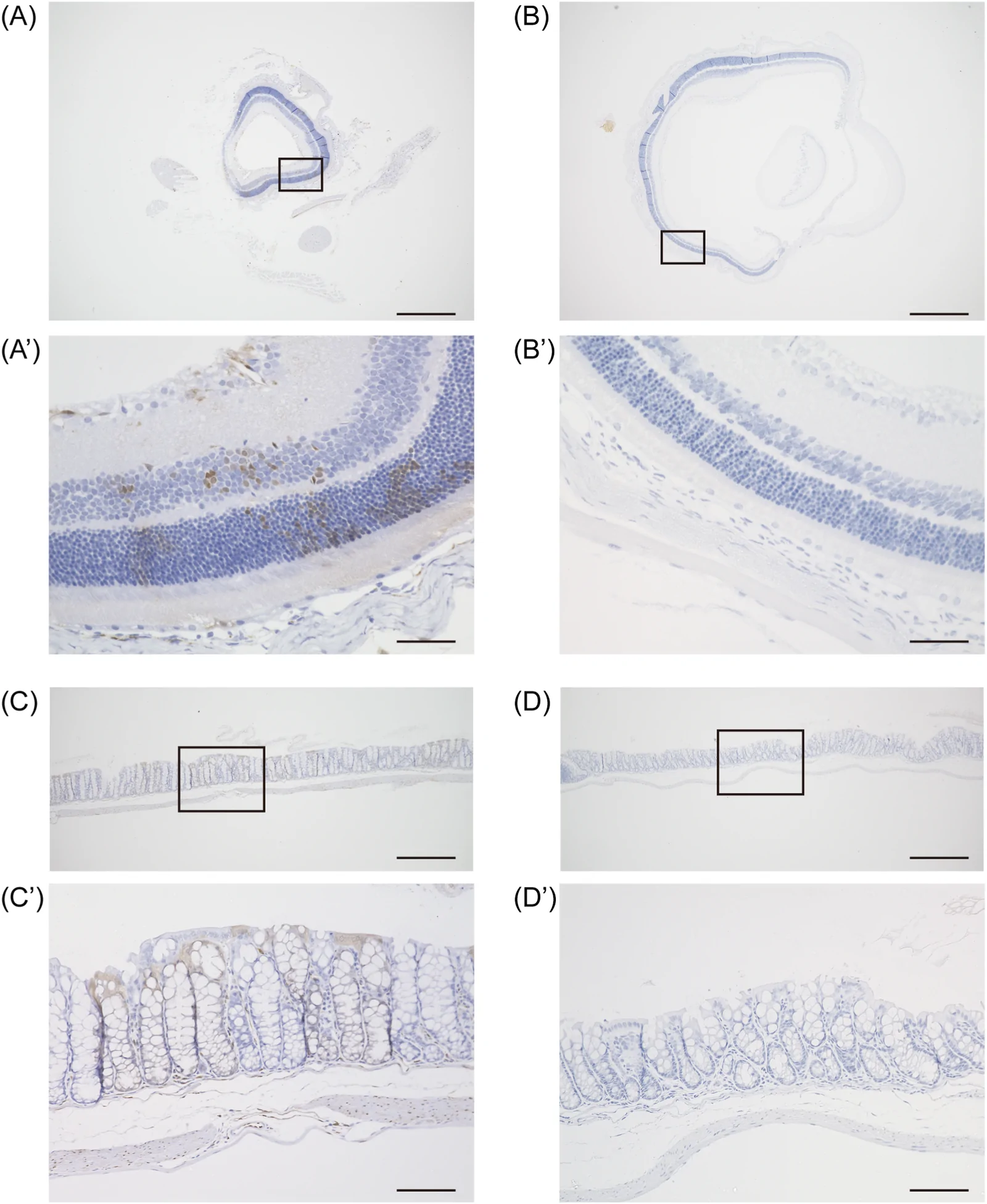
Prior ATRA availability (tDsRed) in the eye and colon. Gal4DBD-RaraLBD; UAS-Cre; R26GRR (A, A′, C, and C′) and UAS-Cre; R26GRR (negative control) samples were stained for tDsRed. (A) A low-magnification image of a Gal4DBD-RaraLBD; UAS-Cre; R26GRR retina. (A′) A high-magnification image of the boxed area in (A). (B) A low-magnification image of a UAS-Cre; R26GRR retina. (B′) A high-magnification image of the boxed area in (B). (C) A low-magnification image of a Gal4DBD-RaraLBD; UAS-Cre; R26GRR colon. (C′) A high-magnification image of the boxed area in (C). (D) A low-magnification image of UAS-Cre; R26GRR colon. (D′) A high-magnification image of the boxed area in (D). Scale bars: 500 μm (A, B, C, and D), 50 μm (A′ and B′), and 100 μm (C′ and D′).

RA signaling acts as a caudalizing factor along the anteroposterior axis during gut development. In the chick embryo, the thyroid develops in the absence of, or at very low levels of, RA, although RA is required for midgut (the future stomach, duodenum, liver, and pancreatic region) development [13]. In contrast to the well-characterized action in chick, RA signaling in mammalian midgut development was unclear. One study showed that RA signaling is present in the liver bud on embryonic day 9.5 in RARE-lacZ mice and is required for hepatic induction from the midgut endoderm [14]. Another study suggested that liver mesothelial cells produce ATRA during development [15]. As RA action throughout the midgut in mammals remains unclear despite extensive examination of the liver, we performed immunohistochemistry for tDsRed in the liver and found tDsRed expression in hepatocytes even distant from the mesothelium (Fig. 3), supporting RA in the liver bud, but not excluding its presence in the liver mesothelium. Current knowledge of ATRA in the stomach is more elusive, as RA production in gastric epithelial cells was expected given the presence of retinol (an ATRA precursor) and RA biosynthesis genes in those cells [16], although direct evidence of ATRA or RA signaling remains lacking. Our immunohistochemistry for tDsRed in the gastric cardia showed tDsRed expression in gastric epithelial cells (Fig. 4), providing the first *in vivo* observation.

**Fig. 3:**
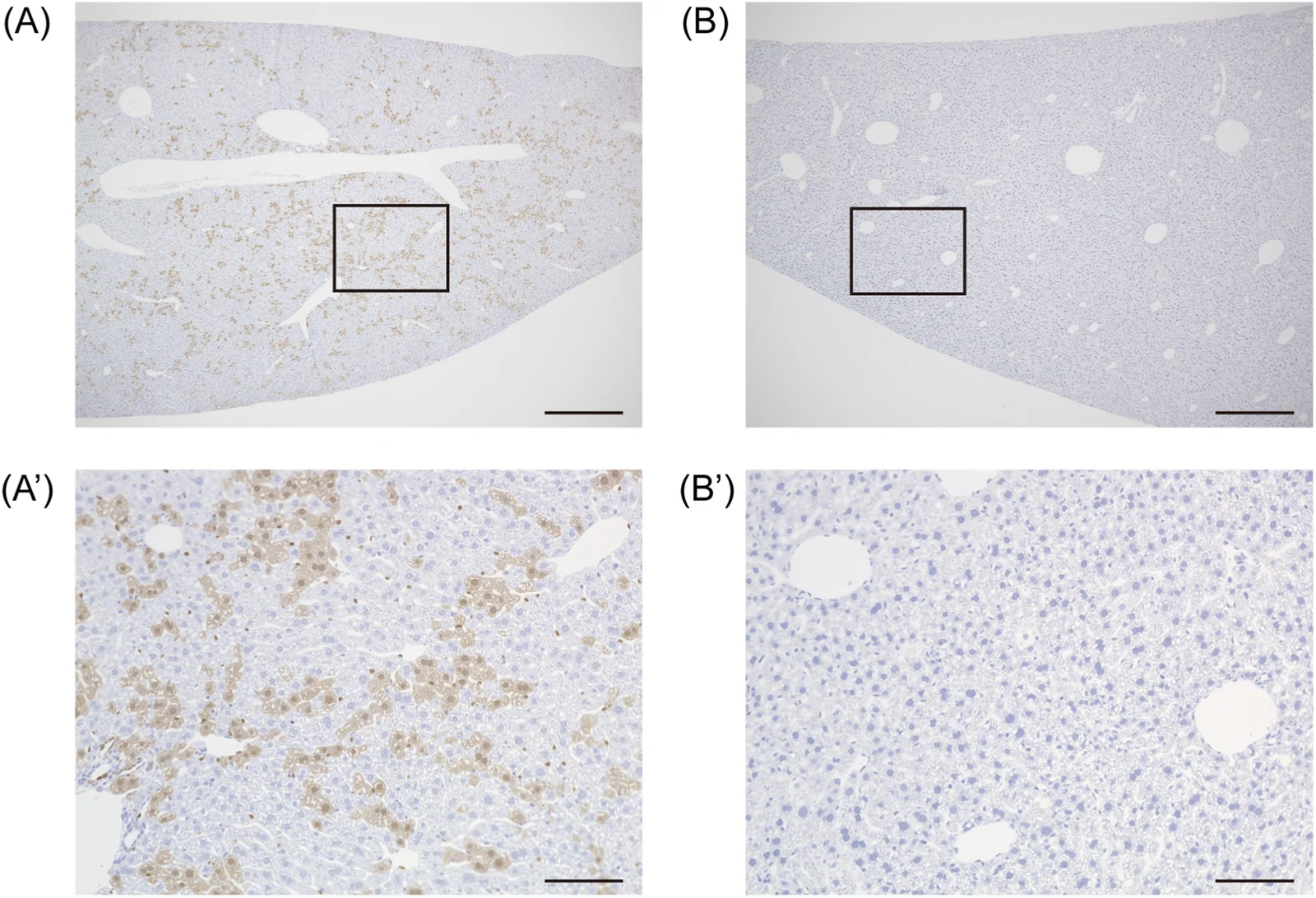
Prior ATRA availability (tDsRed) in the liver. Gal4DBD-RaraLBD; UAS-Cre; R26GRR and UAS-Cre; R26GRR (negative control) samples were stained for tDsRed. (A) A low-magnification image of a Gal4DBD-RaraLBD; UAS-Cre; R26GRR liver. (A′) A high-magnification image of the boxed area in (A). (B) A low-magnification image of a UAS-Cre; R26GRR liver. (B′) A high-magnification image of the boxed area in (B). Scale bars: 500 μm (A and B) and 100 μm (A′ and B′).

**Fig. 4:**
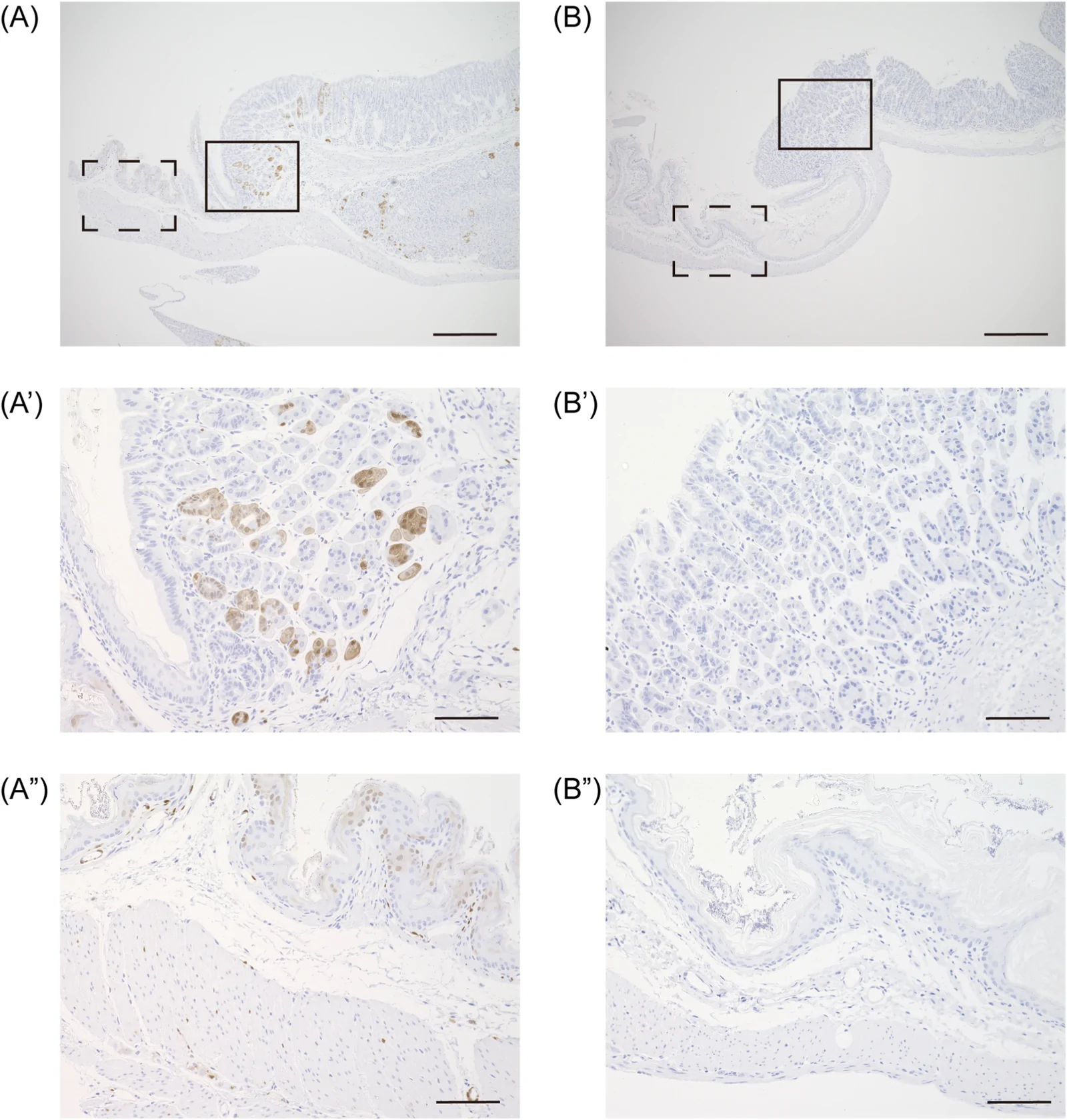
Prior ATRA availability (tDsRed) in the cardia region of the stomach. Gal4DBD-RaraLBD; UAS-Cre; R26GRR and UAS-Cre; R26GRR (negative control) samples were stained for tDsRed. (A) A low-magnification image of a Gal4DBD-RaraLBD; UAS-Cre; R26GRR sample. (A′) A high-magnification image of the boxed area (cardia) in (A). (A″) A high-magnification image of the area surrounded by a dashed line (esophagus) in (A). Note that multilayered squamous epithelial cells indicate the esophagus. (B) A low-magnification image of a UAS-Cre; R26GRR sample. (B′) A high-magnification image of the boxed area (cardia) in (B). (B″) A high-magnification image of the area surrounded by a dashed line (esophagus) in (B). Note that multilayered squamous epithelial cells indicate the esophagus. Scale bars: 500 μm (A and B) and 100 μm (A′, A″, B′, and B″).

Finally, to provide systemic screening results of prior ATRA availability, we performed immunohistochemistry for tDsRed in the heart (Supplementary Material 5), lung (Supplementary Material 6), pancreas (Supplementary Material 7), small intestine (Supplementary Material 8), kidney (Supplementary Material 9), and testis (Supplementary Material 10; n = 1, as Animal ID 2092 was female). For details, see each figure legend.

## 4 Discussion

Signal transduction begins with ligand binding to its receptor, so understanding its regulation requires elucidating three components (ligand, receptor, and signal). In RA signaling, the RARE-lacZ mice helped elucidate signal transduction [7], and many attempts have been made to elucidate the expression of enzymes involved in metabolism or catabolism [1]. Elucidation of the ligand (ATRA) distribution is, however, challenging as it is an unstable small molecule. Our approach is unique in that we focus on the cellular availability of ATRA, as we consider it a biologically meaningful amount *in vivo*. To this aim, we attempted to generate transgenic mice that convert ATRA availability to labelable gene expression (Cre expression).

As suggested in prior literature [8], the Gal4DBD-RaraLBD fusion protein could induce downstream gene expression at a physiological level (30 nM) of ATRA [11] *in vitro* (Supplementary Material 3). Indeed, we observed tDsRed expression that suggests prior ATRA availability in the RA signal-requiring organs, including the eye and colon, in two mice. Unfortunately, we were no longer able to reproduce the result, despite increasing the number of mice examined to 14 from three F1-generation lines. This does not seem to be due to the toxic action of Gal4DBD-RaraLBD because transgene transmission rates were close to the expected value from Mendel's principles of inheritance (50%): 93/183 (50.8%) for line 1711, 28/53 (52.8%) for line 1679, and 21/46 (45.7%) for line 1727. In addition, one limitation in this study is that we did not establish functional Gal4DBD-RaraLBD mouse lines. RT-PCR results suggested that this is partly owing to failure of constitutive expression of the Gal4DBD-RaraLBD fusion protein (Supplementary Material 4). In retrospect, we speculate that this failure could be caused by unfavorable selection of the promoter and enhancer. One study suggested that GC-content significantly influences gene expression levels [17]. Specifically, this study showed that the addition of CpG-island fragments from the HNRPA2B1/CBX3 genomic locus enhanced transgene expression from the CMV promoter/enhancer, which is susceptible to silencing, in mammalian cells. In addition, expression from the CMV promoter/enhancer could be highly dependent on the cell types, compared with EF1A or CAGG promoters [18]. Therefore, refining the promoter would improve Gal4DBD-RaraLBD expression and effectively validate this study's results. This report encourages future studies to design transgenes carefully. In addition, although we sacrificed the F1 generation without examining Gal4DBD-RaraLBD expression because we did not expect low transgene expression, this would have shown whether that risk was possible.

Although this study has such limitations, we report the first attempt to observe ATRA availability. Because our strategy is constitutive expression of an “ATRA-catching” exogenous receptor (Gal4DBD-RaraLBD), the interpretation of the results should differ from that of endogenous gene promoter-driven strategies. Indeed, tDsRed-expressing cells (e.g., retinal neurons, hepatocytes, and gastric epithelial cells) were in line with the literature, suggesting the soundness and potential of our strategy. Overcoming the low expression levels of Gal4DBD-RaraLBD would validate this study's results and further clarify the overlooked availability of ATRA from the embryonic stage to adulthood, which might be associated with human development and health.

## 5 Conclusion

We reported the first observation of ATRA availability at single-cell resolution in mice, although further investigation is required to validate the result.

## CRediT authorship contribution statement

**Masaharu Yoshihara:** Writing – original draft, Visualization, Project administration, Methodology, Investigation, Funding acquisition, Formal analysis, Data curation, Conceptualization. **Yoshitoku Watabe:** Writing – original draft, Investigation. **Baoshuo Ning:** Investigation. **Hayate Suzuki:** Resources. **Seiya Mizuno:** Resources. **Satoru Takahashi:** Supervision.

## Ethics declaration

This study was conducted in accordance with the following guidelines for animal welfare and/or reporting: Animal experiments were conducted in accordance with the institutional or national guidelines of the University of Tsukuba and Japan. This study was approved by the University of Tsukuba. (Approval No. 24-411, 25-160, 26-307 (animal experiments), and 240208 (DNA experiments))

## Declaration of competing interest

The authors declare the following financial interests/personal relationships which may be considered as potential competing interests: Masaharu Yoshihara reports financial support was provided by Japan Society for the Promotion of Science. If there are other authors, they declare that they have no known competing financial interests or personal relationships that could have appeared to influence the work reported in this paper.

## Figures and Tables

**Figure:**
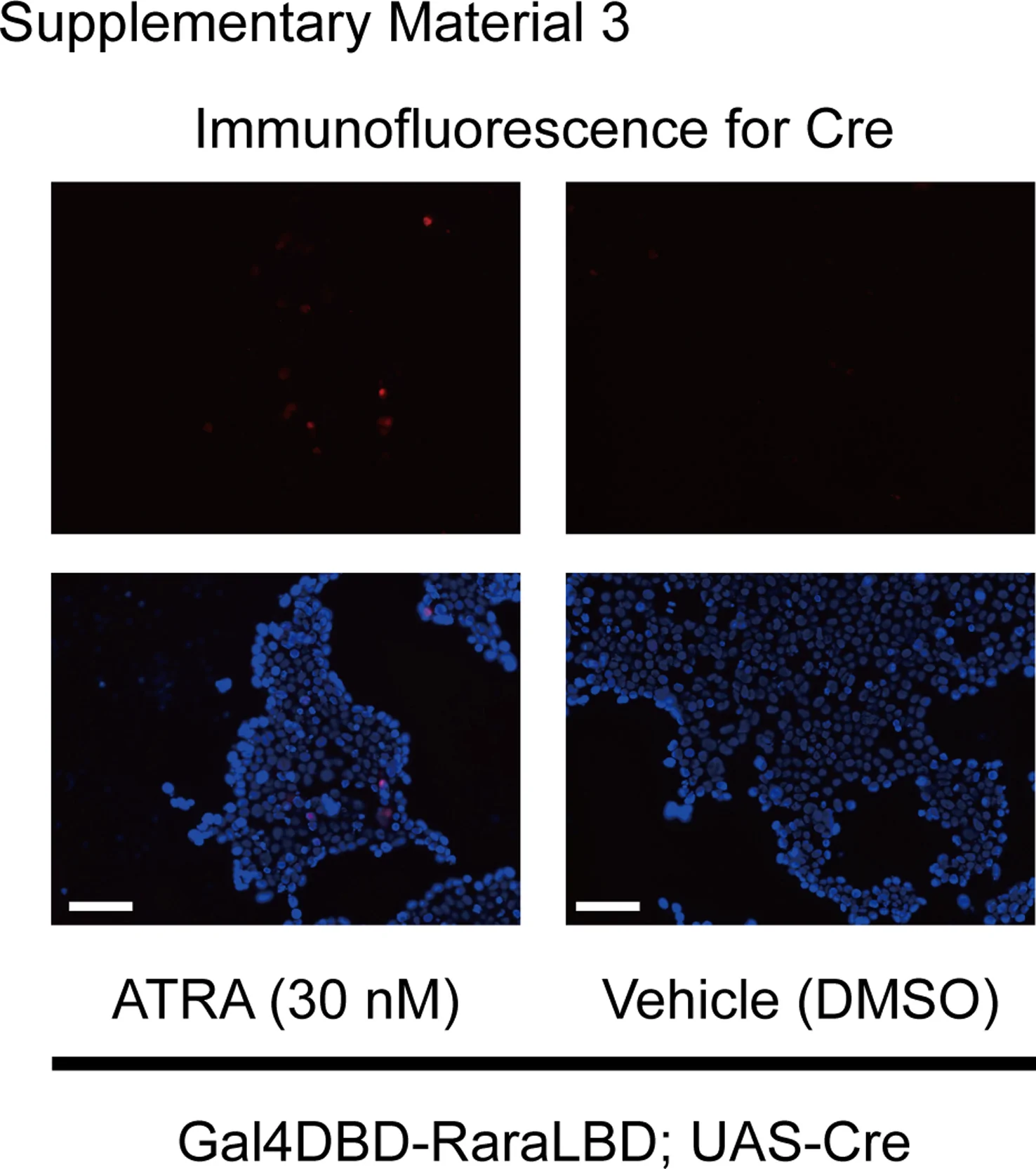
Supplementary Material 4 R T-PCR analysis of transgene expression. The upper table shows sample information (Mouse ID, Genotype, and Organ) and Ct values determined by the SDM method. For brevity, Gal4DBD-RaraLBD is referred to as Gal4Rara. The lower table shows the validity of PCR primers for Gal4DBD-RaraLBD, as assessed by a dilution series of the pcDNA_Gal4DBD-RaraLBD plasmid. In both tables, hyphens indicate low expression from which Ct values cannot be calculated. N.A.: Not Applicable.

**Figure:**
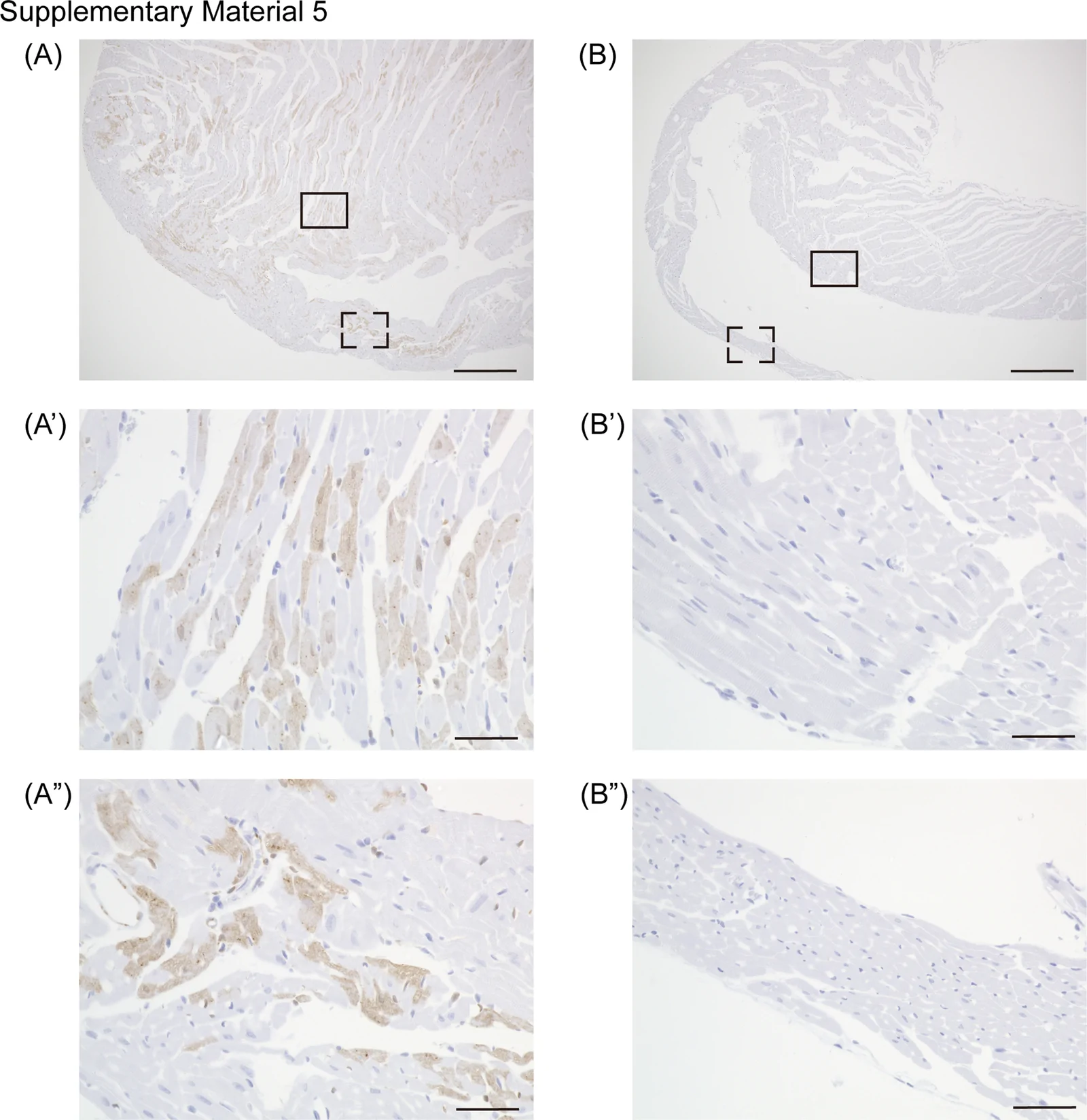
Supplementary Material 5 Immunohistochemistry for tDsRed in the heart. (A) A low-magnification image of a Gal4DBD-RaraLBD; UAS-Cre; R26GRR mouse heart. (A′) A high-magnification image of the boxed area in (A) (the ventricular septum). (A″) A high-magnification image of the area surrounded by a dashed line in (A) (the right ventricle). tDsRed expression was observed in the ventricular cardiomyocytes, which is consistent with the fact that the ventricular cardiomyocytes were affected by impaired embryonic ATRA synthesis [19]. (B) A low-magnification image of a UAS-Cre; R26GRR mouse heart. (B′) A high-magnification image of the boxed area in (B) (the ventricular septum). (B″) A high-magnification image of the area surrounded by a dashed line in (B) (the right ventricle). Scale bars = 500 μm (A and B) and 50 μm (A′, A″, B′, and B″).

**Figure:**
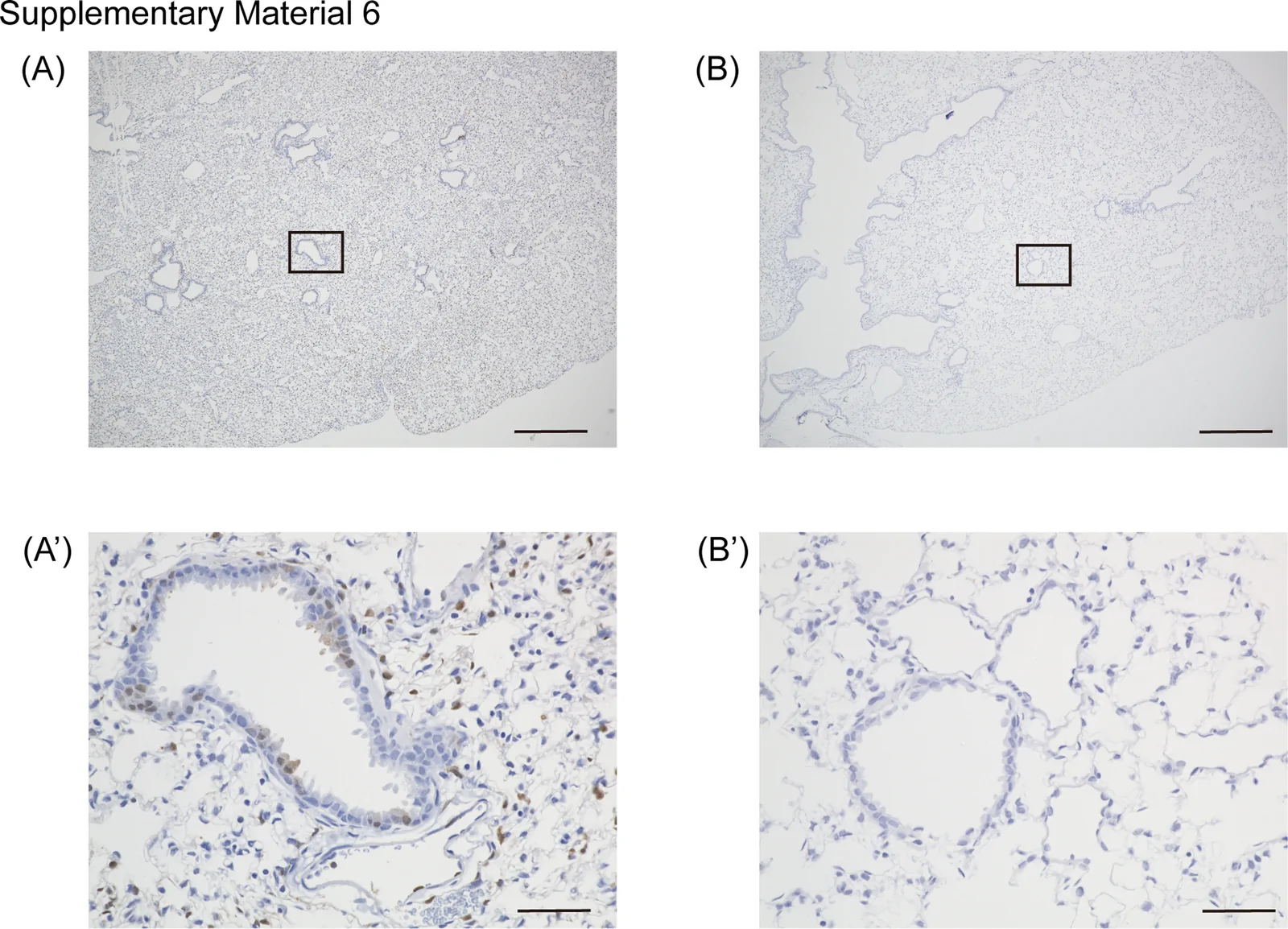
Supplementary Material 6 Immunohistochemistry for tDsRed in the lung. Gal4DBD-RaraLBD; UAS-Cre; R26GRR and UAS-Cre; R26GRR (negative control) samples were stained for tDsRed. (A) A low-magnification image of a Gal4DBD-RaraLBD; UAS-Cre; R26GRR lung. (A′) A high-magnification image of the boxed area in (A). tDsRed was observed in bronchial epithelial cells, consistent with developing lung epithelial cells receiving RA signaling in mouse day 10 embryos [20]. (B) A low-magnification image of a UAS-Cre; R26GRR lung. (B′) A high-magnification image of the boxed area in (B). Scale bars: 500 μm (A and B) and 50 μm (A′ and B′).

**Figure:**
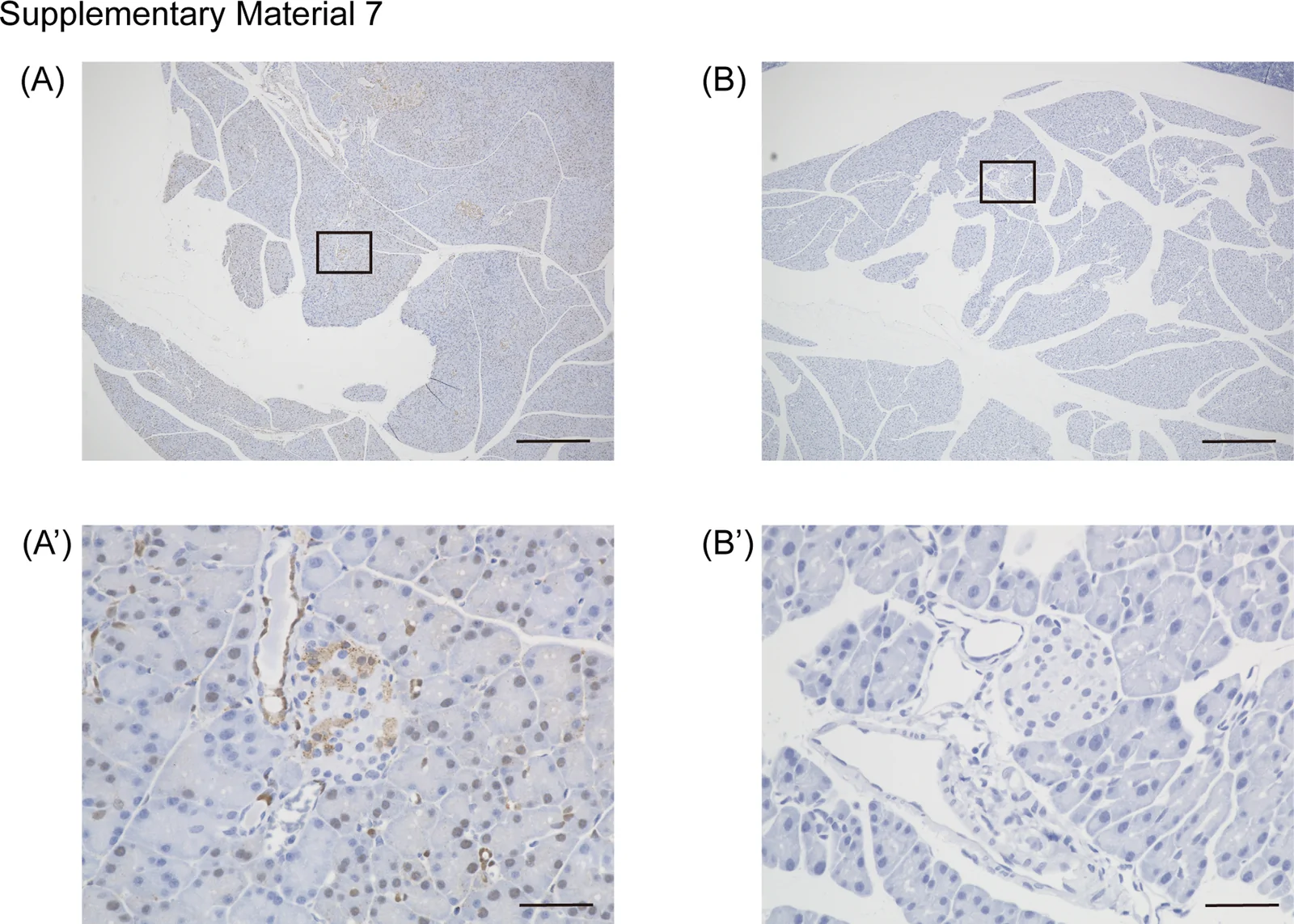
Supplementary Material 7 Immunohistochemistry for tDsRed in the pancreas. Gal4DBD-RaraLBD; UAS-Cre; R26GRR and UAS-Cre; R26GRR (negative control) samples were stained for tDsRed. (A) A low-magnification image of a Gal4DBD-RaraLBD; UAS-Cre; R26GRR pancreas. (A′) A high-magnification image of the boxed area in (A). tDsRed expression was observed in both acinar and islet cells. Note that this is consistent with the fact that dorsal pancreas development was affected by impaired embryonic ATRA synthesis [21]. (B) A low-magnification image of a UAS-Cre; R26GRR pancreas. (B′) A high-magnification image of the boxed area in (B). Scale bars: 500 μm (A and B) and 50 μm (A′ and B′).

**Figure:**
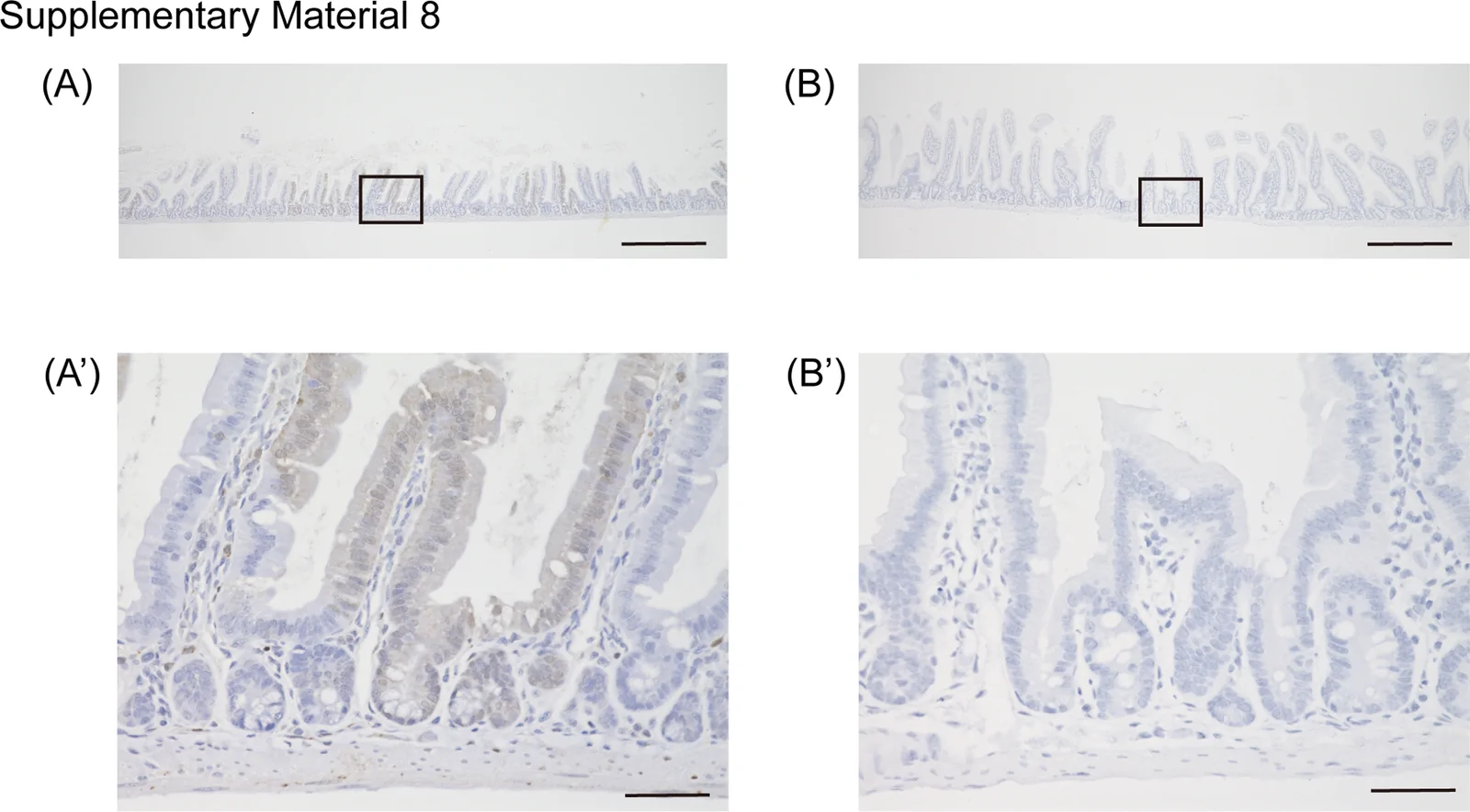
Supplementary Material 8 Immunohistochemistry for tDsRed in the small intestine. Gal4DBD-RaraLBD; UAS-Cre; R26GRR and UAS-Cre; R26GRR (negative control) samples were stained for tDsRed. (A) A low-magnification image of a Gal4DBD-RaraLBD; UAS-Cre; R26GRR small intestine. (A′) A high-magnification image of the boxed area in (A). tDsRed expression was observed in the epithelial cells from the crypt to the tip of the villi, consistent with the fact that small intestinal epithelial cells were suggested to receive RA signaling [22]. (B) A low-magnification image of a UAS-Cre; R26GRR intestine. (B′) A high-magnification image of the boxed area in (B). Scale bars: 500 μm (A and B) and 50 μm (A′ and B′).

**Figure:**
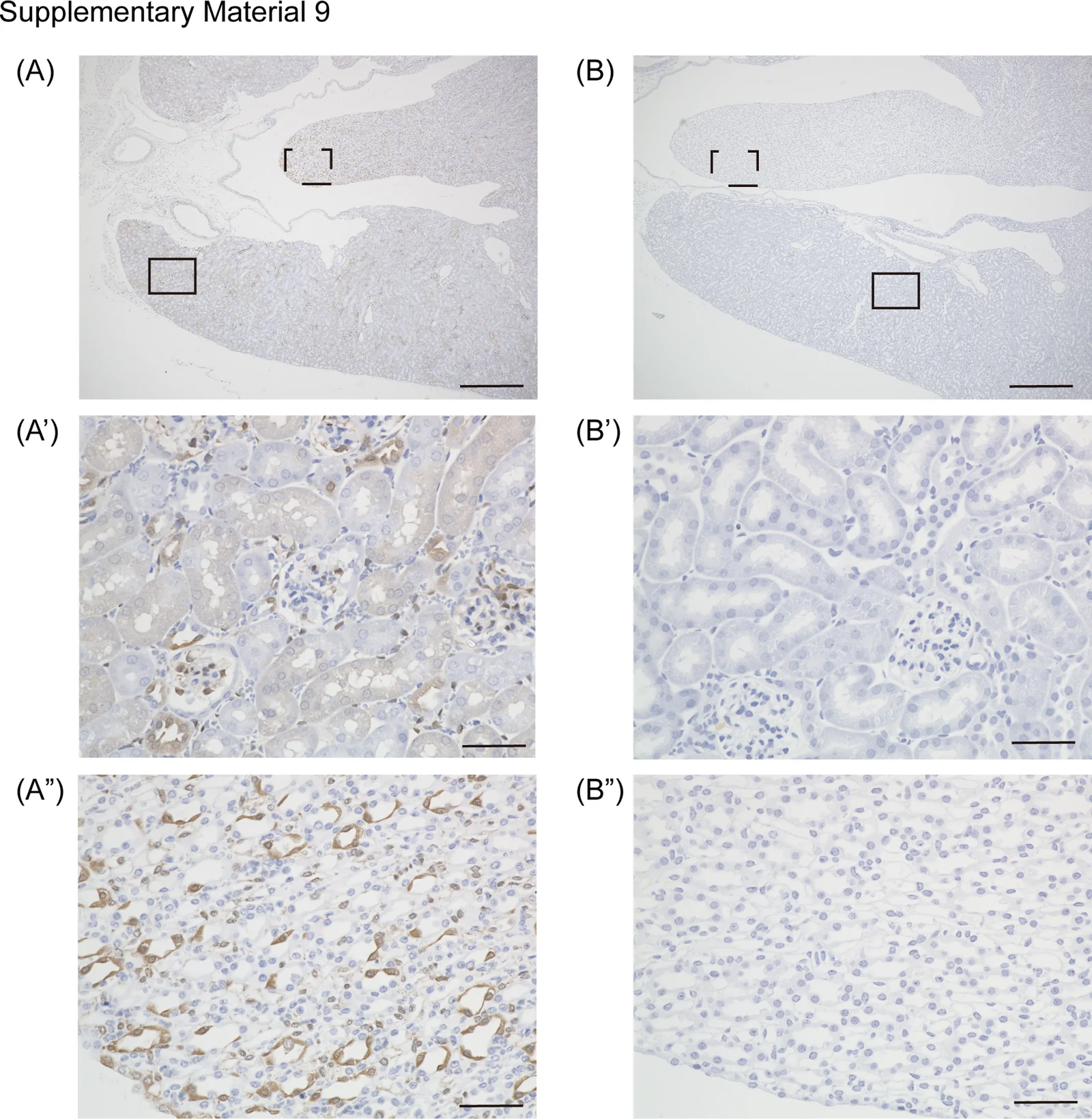
Supplementary Material 9 Immunohistochemistry for tDsRed in the kidney. Gal4DBD-RaraLBD; UAS-Cre; R26GRR and UAS-Cre; R26GRR (negative control) samples were stained for tDsRed. (A) A low-magnification image of a Gal4DBD-RaraLBD; UAS-Cre; R26GRR kidney. (A′) A high-magnification image of the boxed area (cortex) in (A). (A″) A high-magnification image of the medulla area surrounded by a dashed line in (A). tDsRed expression was observed in both the proximal and distal tubule epithelial cells in the cortex (A′) and epithelial cells in the medulla (A″), consistent with the fact that ureteric bud cells (the precursor of the epithelial cells from renal distal tubules to the bladder) receive RA signaling [23]. (B) A low-magnification image of a UAS-Cre; R26GRR kidney. (B′) A high-magnification image of the boxed area (cortex) in (B). (B″) A high-magnification image of the medulla area surrounded by a dashed line in (B). Scale bars: 500 μm (A and B) and 50 μm (A′, A″, B′, and B″).

**Figure:**
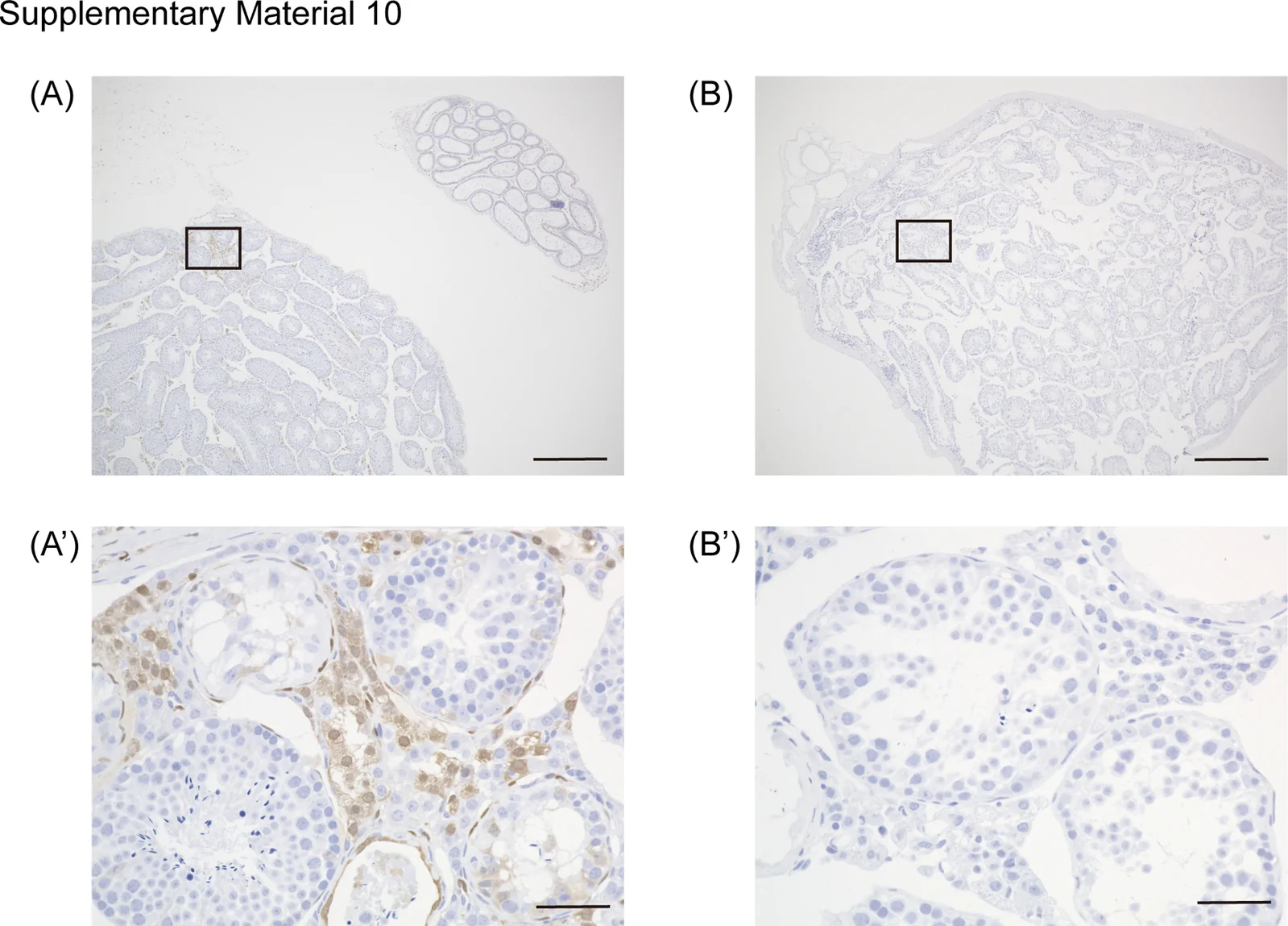
Supplementary Material 10 Immunohistochemistry for tDsRed in the testis. (A) A low-magnification image of a Gal4DBD-RaraLBD; UAS-Cre; R26GRR testis. (A′) A high-magnification image of the boxed area in (A). tDsRed expression was observed mainly in testicular stromal cells. Although the type of tDsRed-expressing cells in the seminiferous tubules is unclear, it is interesting to note that RA signaling in Sertoli cells supports sperm maturation [24]. In addition, RA signaling in Leydig cells maintains the blood-testis barrier [25]. (B) A low-magnification image of a UAS-Cre; R26GRR testis. (B′) A high-magnification image of the boxed area in (B). Scale bars: 500 μm (A and B) and 50 μm (A′ and B′).

## Data Availability

Data will be made available on request.

## List of abbreviations

*All-trans-*retinoic acid (ATRA), bovine growth hormone poly A sequence (bGH pA), cytomegalovirus (CMV), DNA-binding domain (DBD), Gal4 DNA-binding domain (Gal4DBD), ligand-binding domain (LBD), retinoic acid receptor alpha ligand-binding domain (RaraLBD), retinoic acid (RA), reverse transcription polymerase chain reaction (RT-PCR), SV40 nuclear localization sequence (NLS), tandem DsRed (tDsRed), upstream activating sequence (UAS).

## Appendix A Supplementary data

Multimedia component 1 Supplementary Material 1 Plasmid map of the transgene generated in this study. This map shows the transgene elements and restriction sites. The transgene elements from the human β-globin/IgG chimeric intron to RaraLBD were incorporated into the pcDNA3 plasmid at the EcoR1 and Not1 restriction sites.

Multimedia component 2 Supplementary Material 2 DNA sequence of the transgene. This file contains the DNA sequence of the pcDNA3_Gal4DBD-RaraLBD plasmid.

Multimedia component 3 Supplementary Material 3 Transgene functionality. Immunofluorescence for Cre recombinase. HEK293T cells were transfected with the pcDNA3_Gal4DBD-RaraLBD plasmid and UAS-Cre plasmid, followed by administration of either 30 nM RA or vehicle (DMSO). Representative immunofluorescent images for Cre recombinase are shown (above: Cre expression, below: overlaid images with nuclear staining). Scale bars: 100 μm.
