# Supplementary material for: Cellular availability of all-trans-retinoic acid in mice: a pilot study
Source: Biochem Biophys Rep. 2026 Oct 15;48:102839. doi: 10.1016/j.bbrep.2026.102839 (PMC13694855; doi:10.1016/j.bbrep.2026.102839)

Supplementary Material 1

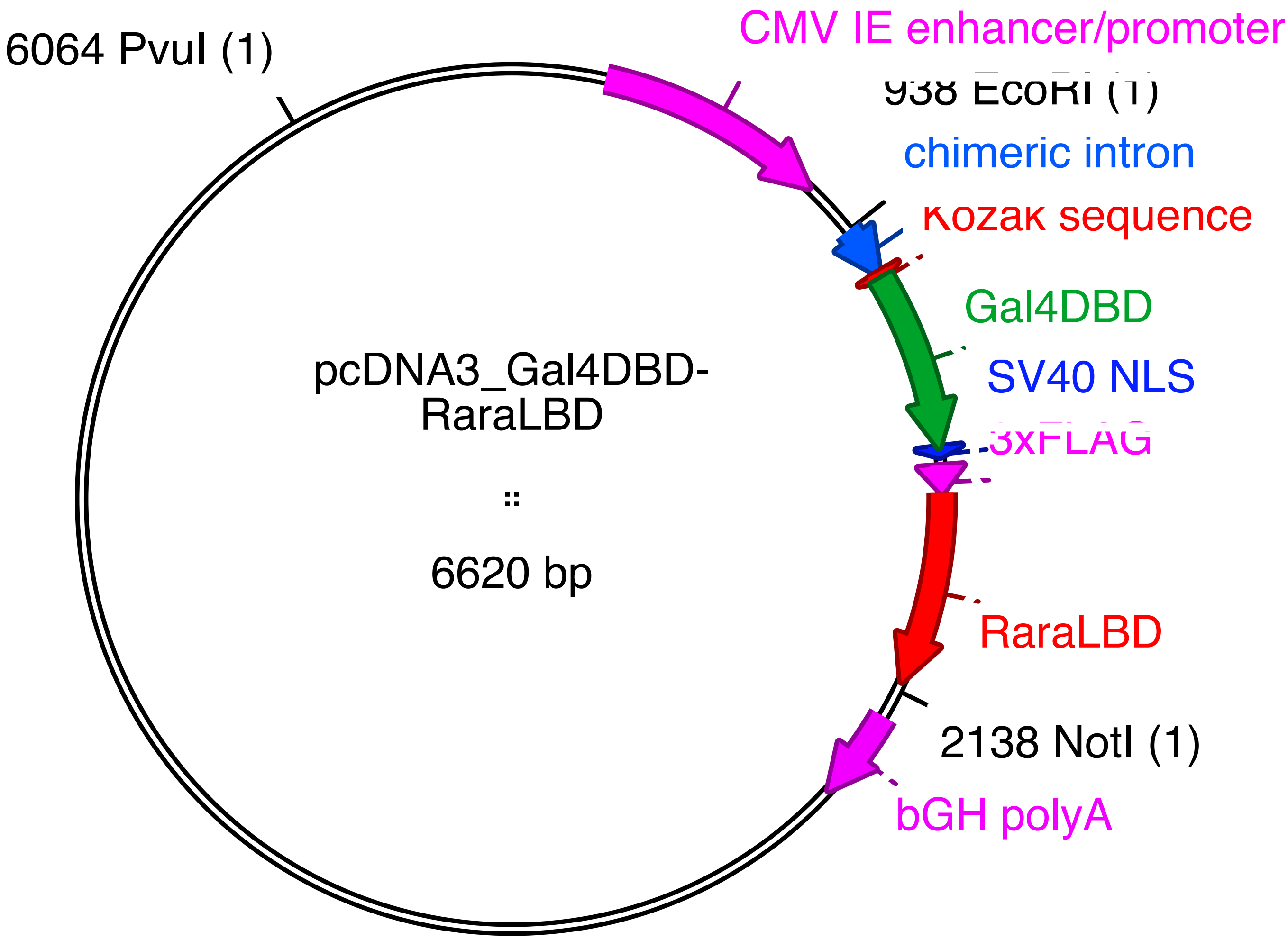

Supplement: Supplementary file 1 [file mmc1.pdf]
