# Supplementary material for: Cellular availability of all-trans-retinoic acid in mice: a pilot study
Source: Biochem Biophys Rep. 2026 Oct 15;48:102839. doi: 10.1016/j.bbrep.2026.102839 (PMC13694855; doi:10.1016/j.bbrep.2026.102839)
Supplement: Supplementary file 2 [file mmc3.pdf]

Supplementary Material 4

RT-PCR results (Ct value by SDM method)

Mouse ID	Genotype	Organ	Gal4Rara Duplicate 1	Gal4Rara Duplicate 2	HPRT Duplicate 1	HPRT Duplicate 2
1652	Wild-type	Eye	37.82	-	26.99	27.35
1653	Wild-type	Eye	37.63	36.33	26.69	27.13
1832	Wild-type	Eye	38.06	-	25.41	25.42
1887	Gal4Rara	Eye	38.3	-	25.68	25.69
1892	Gal4Rara	Eye	-	36.91	27.2	27.32
2089	Gal4Rara	Eye	-	37.47	25.4	25.84
1652	Wild-type	Colon	-	34.98	26.22	26.22
1653	Wild-type	Colon	-	-	26.05	25.97
1832	Wild-type	Colon	37.86	-	25.41	25.3
1887	Gal4Rara	Colon	-	37.28	25.51	25.54
1892	Gal4Rara	Colon	-	-	25.08	25.1
2089	Gal4Rara	Colon	-	36.63	24.59	24.59

PCR Template	Gal4Rara Duplicate 1	Gal4Rara Duplicate 2	HPRT Duplicate 1	HPRT Duplicate 2
Gal4DBD-RaraLBD Plasmid (1 ng)	11.95	12.09	N.A.	N.A.
Gal4DBD-RaraLBD Plasmid (0.1 ng)	15.46	15.57	N.A.	N.A.
Gal4DBD-RaraLBD Plasmid (0.01 ng)	18.27	18.58	N.A.	N.A.
Gal4DBD-RaraLBD Plasmid (0.001 ng)	22.41	22.43	N.A.	N.A.
DDW (no DNA)	-	-	-	37.33
